# Fistule œso-trachéale secondaire à une intubation trachéale prolongée

**DOI:** 10.11604/pamj.2015.20.388.6758

**Published:** 2015-04-20

**Authors:** Abdelkarim Shimi, Mohammed Khatouf

**Affiliations:** 1 Service de Réanimation Polyvalente A1, CHU Hassan II, Faculté de Médecine et de Pharmacie, Université Sidi Mohamed ben Abdallah, Fès, Maroc

**Keywords:** Fistule œso-trachéale, intubation trachéale prolongée, tracheotomie

## Image en medicine

Patient âgé de 27 ans, hospitalisé au service de réanimation chirurgicale pour prise en charge d'un traumatisme crânien grave avec un score de Glasgow initiale à 4. Le patient était trachéotomisé à J6 de son admission. AJ45, le diagnostic d'une fistule oeso-trachéale a été évoqué devant un tableau clinique fait de pneumopathies a répétition avec présence de débris alimentaire au niveau du liquide d'aspiration trachéale. la fibroscopie œsophagienne a confirmé la présence de la fistule(A), et le scanner thoracique a montré une large communication entre la paroi postéro-latérale gauche de la trachée et la lumière œsophagienne (B). La fistule œso-trachéale est une complication rare (incidence 0,5 %) de l'intubation trachéale prolongée. Les causes à l'origine de cette complication sont la lésion per opératoire de la paroi postérieure de la trachée, une mauvaise position de la canule notamment sa coudure, une mauvaise gestion du ballonnet en particulier des pressions, une sonde gastrique de gros calibre ou trop rigide, une infection fongique de l'œsophage et la dénutrition du patient. La prise en charge thérapeutique de ces patients va nécessiter, dans un premier temps, d'arrêter l'inhalation du liquide gastrique à travers la fistule et, de mettre au repos l'œsophage. Le traitement chirurgical est indiqué dans un 2^ème^ temps. Les récidives sont possibles et la mortalité n'est pas négligeable (6,3 à12, 5%).

**Figure 1:**
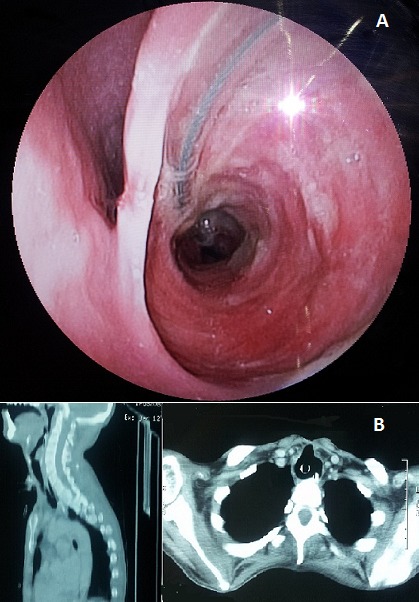
A) endoscopie œsophagienne: mise en évidence d'une fistule œso-trachéale d'environ 2cm; B) TDM thoracique avec injection intraveineuse du produit de contraste iodé et Reconstruction 2D dans le plan sagittal montrant une fistule oeso-trachéale avec une large communication entre la paroi postéro-latérale gauche de la trachée et la lumière œsophagienne

